# Gestational hypertension and childhood atopy: a Millennium Cohort Study analysis

**DOI:** 10.1007/s00431-021-04012-3

**Published:** 2021-03-26

**Authors:** Ian Henderson, Siobhan Quenby

**Affiliations:** 1 Warwick Medical School, The University of Warwick, Coventry, CV4 7AL UK; 2 University Hospital Coventry, Coventry, CV2 2DX UK

**Keywords:** Atopy, Tolerance, Pre-eclampsia, Asthma, Hay fever, Eczema

## Abstract

Gestational hypertension may confer risk of atopic disease in offspring through a direct biological mechanism, but another possibility is that risk is mediated through complications of pregnancy. To explore these associations, we conducted an analysis of a nationally representative birth cohort based in the UK involving children born 2000–2002. We included 12,450 mother-child pairs. We used logistic regression to estimate the association between hypertensive disease and asthma, hay fever, or eczema by age 5, and parentally reported early wheeze and severe wheeze. Mediation by gestation at delivery and caesarean delivery was explored using causal mediation analysis. Odds ratios (95% CI) for gestational hypertension and childhood asthma, hay fever, and eczema were 1.32 (1.09, 1.59), 1.22 (0.97, 1.55), and 1.12 (0.96, 1.32) respectively, adjusted for confounding. The population attributable fractions were 2.4% (1.0–3.8%), 0.9% (−0.3% to 2.1%), and 1.8% (0.0–3.7%), respectively. Accounting for mediation by gestational age and caesarean delivery, odds ratios (95% CI) for the potential direct effects of gestational hypertension were 1.21 (0.97, 1.50), 1.17 (0.91, 1.49), and 1.11 (0.94, 1.31) for the same.

*Conclusion*: Gestational hypertension was weakly positively associated with asthma and this was partly mediated by earlier delivery. Only a small proportion of early childhood asthma was attributable to gestational hypertensive disease in this representative UK-based birth cohort.

**Table:** 

**What is known:** *• Gestational hypertension has been shown to be an inconsistent risk factor for the atopic diseases.* *• The in utero immune environment may modify the risk of atopy in offspring; alternatively, complications of pregnancy including caesarean delivery and prematurity may explain an association between hypertensive disease and atopy.*	
**What is new:** *• Self-reported gestational hypertension was a weak risk factor for asthma and wheeze in the Millennium Cohort Study.* *• Part of the association between gestational hypertensive disease and asthma was explained by earlier delivery.*

**What is known:**

*• Gestational hypertension has been shown to be an inconsistent risk factor for the atopic diseases.*

*• The in utero immune environment may modify the risk of atopy in offspring; alternatively, complications of pregnancy including caesarean delivery and prematurity may explain an association between hypertensive disease and atopy.*

**What is new:**

*• Self-reported gestational hypertension was a weak risk factor for asthma and wheeze in the Millennium Cohort Study.*

*• Part of the association between gestational hypertensive disease and asthma was explained by earlier delivery.*

## Introduction

Atopy describes the tendency to develop allergic diseases like atopic eczema, asthma, hay fever, and food allergy, which often occur in those with elevated immunoglobulin E (IgE) levels. A simplified blueprint of atopy includes genetic predisposition to epithelial barrier dysfunction, aberrant antigen exposure, and atopic activation [1, 2]. Fillagrin gene mutations, pattern receptor gene mutations, and cytokine-related gene mutations have been implicated [1–4]. As our knowledge of the genetic determinants of atopic disease has advanced, so has our knowledge that atopic disease encompasses an array of allergic phenotypes in which elevated IgE is a common component [2] and the notion of the “atopic march” has been complicated [5, 6]. Environmental determinants of specific atopic phenotypes may include in utero exposures, particularly those leading to immune dysfunction. Pregnancy-induced hypertension (PIH) is the new onset of hypertension in pregnancy after 20 weeks of gestation. The disease may progress to glomerular endotheliosis and increased vascular permeability which produces the additional characteristic proteinuria of pre-eclampsia (PET). Dysregulation of communication at the maternal decidual and fetal trophoblastic interface is recognized as an antecedent of PET, which may reduce immune tolerance of the fetus [7] and produce inflammation characterized by decreased regulatory T-cells and anti-inflammatory cytokines [8, 9], features of both PET and atopic disease [10–13]. PET may be a risk factor for atopy in offspring; however, complications of pregnancy as a result of PET, like earlier delivery or caesarean delivery, may equally confer risk. Determining the direction and strength of the association between gestational hypertensive disease and individual atopic diseases may help to characterize the atopic phenotypes and inform pre-conception or antenatal prevention strategies.

## Objective

The aim is to investigate the role of gestational hypertension in the development of early childhood atopic disease and to characterize this association further by considering the mediating role of gestational age and caesarean delivery which may lie on the causal path between gestational hypertension and atopic disease.

## Methods

### Study population

**Fig. 1:**
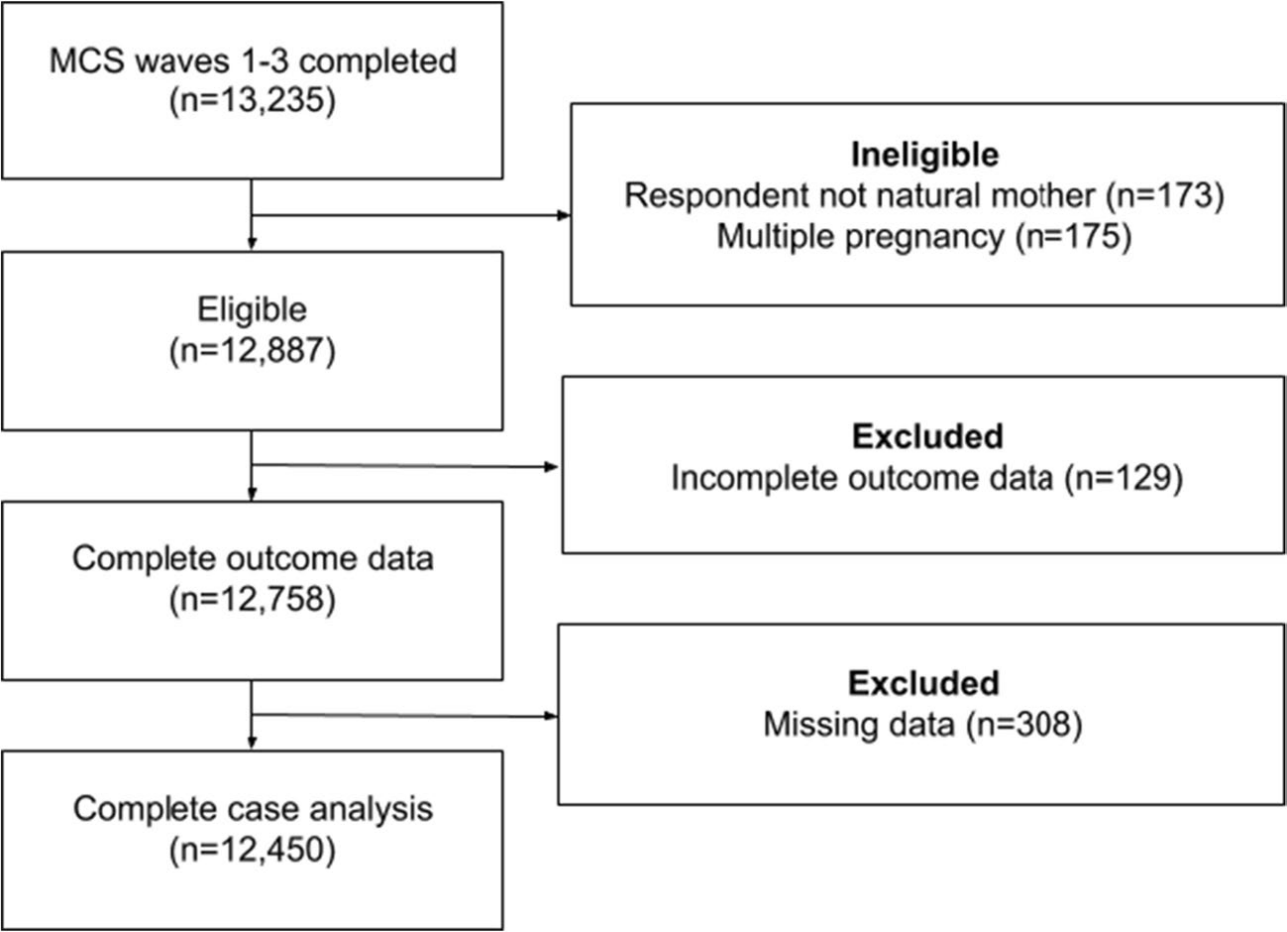
Flow diagram of participants according to eligibility and exclusion criteria. *MCS 1-3*, Millennium Cohort Study survey waves 1–3

The Millennium Cohort Study (MCS) is a UK-based longitudinal study of the socioeconomic circumstance, growth, and development of children born 1 September 2000–31 August 2001 [14]. Data collection is ongoing with the first three waves collected at approximately 9 months [15], 3 years [16], and 5 years of age [17]. The sample frame incorporated children resident in the UK at 9 months of age whose parents were eligible to receive childhood tax benefit. Although near-universal eligibility, asylum seekers were ineligible for childhood tax benefit [14] and undocumented households were also excluded. The survey was clustered at the electoral ward level with UK-wide coverage, and with oversampling of deprived and more ethnically diverse wards such that on baseline sampling at the first wave, 82% of cohort members were White, 2.5% Indian, 4.8% Pakistani, 2% Bangladeshi, 1.3% Black Caribbean, and 2% Black African with the remaining 3% of mixed ethnicity [14]. At wave 1, the achieved sample was 18,552 families from an issued sample of 21,180; at wave 2, 15,590 families were achieved from 19,244 issued including 1389 new families not issued at wave 1; at wave 3, 15,246 families were achieved from 18,528 issued [14]. Included in the present analysis were mother-child pairs with responses for the first three surveys, where baseline survey was completed by the natural mother following a singleton pregnancy and subsequent surveys by either natural parent. The study flow diagram for the current analysis is shown in Fig. 1.

### Exposure

In the MCS, women were asked whether they experienced “any illnesses or other problems during [their] pregnancy that required medical attention or treatment?” If answered affirmatively, they were asked whether they suffered “raised blood pressure, eclampsia/ pre-eclampsia or toxaemia” during pregnancy [18]. The exposure was gestational hypertension, defined as a self-reported history of PIH or PET, which were combined in the MCS wave 1 questionnaire.

### Outcomes

Outcomes were parentally reported eczema, asthma, or hay fever at the age 5 wave [18]. Secondary outcomes were parentally reported symptomatic outcomes of early wheeze (wheeze under 3 years of age) and severe wheeze (≥4 attacks of wheeze or ≥ 1 night of sleep disturbance secondary to wheeze or wheeze that affects speech within the last 12 months at age 5), as defined by the International Study of Asthma and Allergies in Childhood (ISAAC) criteria [19].

### Potential confounders

Measured confounders considered included maternal covariables of age at delivery (<25 years, 25–29 years, 30–34 years, ≥35 years), ethnicity (Black, South Asian, White, Other), highest educational attainment (no recognized level, foundation diploma, higher diploma, advanced diploma, certificate of higher education, foundation degree, bachelor’s degree, postgraduate degree), history of asthma (yes, no), hay fever (yes, no), eczema (yes, no), parity (primiparous, multiparous), and smoking status during pregnancy (non-smoker, <10 cigarettes, ≥10 cigarettes per day). Women were asked about any longstanding illness, and diagnoses were grouped by ICD-10 code [18]; adjustment was made for a history of essential hypertension on report of essential hypertension by ICD-10 code I10 (yes, no). Household covariables included OECD equivalized household income quintile. Infant covariables were birth weight adjusted for gestation and sex, to account for sex differences and to avoid biasing estimates for gestation due to collinearity. We also included infant sex, which has been associated with atopy as well as gestational hypertensive disease in a recent meta-analysis which included several large population studies [20].

### Potential mediators

Potential mediators considered were gestation at birth (days) and caesarean delivery (vaginal, caesarean).

### Statistical analysis

MCS survey weights were used throughout the statistical analysis to account for both stratified cluster sampling and for non-response attrition bias. Descriptively, unweighted counts and weighted proportions of maternal and infant characteristics were calculated according to exposure for categorical variables. Weighted medians and interquartile ranges were calculated for non-normally distributed continuous variables, and weighted mean z-scores and 95% confidence intervals (CIs) were calculated for birth weight adjusted for gestation and sex using the Intergrowth calculator [21, 22]. The functional form of continuous variables was examined. Odds ratios and 95% CIs were calculated for each outcome using multiple logistic regression with model selection according to literature review. A partially adjusted model was adjusted for maternal age, education and ethnicity, household income, maternal history of atopic diseases, parity, smoking status during pregnancy, essential hypertension, infant sex, and birth weight adjusted for gestation and sex. Estimates are with reference to their respective specific disease-free populations. A fully adjusted model was additionally adjusted for gestational age and caesarean delivery. Mediation by gestational age and caesarean delivery was explored in causal mediation analysis. Causal mediation analysis was completed using the third-party Stata package ‘khb’ based on the method outlined by Breen et al. [23]. The total, direct, and combined and individual indirect effects of gestational age and caesarean delivery were calculated with adjustment for the above confounders. Population attributable fractions were calculated using the Stata package ‘punaf’ based on the method outlined by Greenland and Drescher [24] to estimate the proportion of cases of the primary outcomes secondary to gestational hypertension, under the assumption of a causal relationship. All analyses were completed using Stata version 16 (StataCorp, 2019). Tests were considered significant at a two-tailed *p*-value <0.05.

## Results

There were 13,235 mother-child pairs with responses at all three survey waves. Of these, 175 were ineligible due to a multiple pregnancy and 173 were ineligible due to response from someone other than the natural mother at wave 1 or other than a natural parent at waves 2 and 3. Subsequently, 12,887 mother-child pairs were eligible and of these, 129 were missing outcome data and 308 were missing data on any covariable and were excluded. Data were missing for <1% of all participants across all covariables except for parity, which had missingness of 1.1%, leading to the exclusion of 3.4% of eligible participants in total. We therefore proceeded to a complete case analysis of 12,450 mother-child pairs of which 950 mothers (7.7%) had gestational hypertensive disease. Among offspring, 4242 (35%) suffered from eczema, 1857 (15%) asthma, and 1342 (10%) hay fever (weighted proportions) whilst 3805 (31%) were symptomatic of early wheeze and 924 (7.3%) severe wheeze. All outcomes were commoner among offspring to women with gestational hypertension, as shown in Table 1. Women with gestational hypertension tended to have higher levels of education and household income, and a greater proportion suffered from maternal asthma, hay fever, or eczema. The caesarean rates were higher and median gestational age lower for women with gestational hypertension. Unadjusted, there was strong evidence that gestational hypertension was positively associated with early wheeze (OR 1.34 [95% CI 1.14–1.58], *p*=0.001), asthma (OR 1.35 [95% CI 1.12–1.62], *p*=0.002), hay fever (OR 1.36 [95% CI 1.08–1.71], *p*=0.008), and eczema (OR 1.19 [95% CI 1.02–1.40], *p*=0.03), and there was weaker evidence of an association with severe wheeze (OR 1.31 [95% CI 0.99–1.72], *p*=0.06). After adjustment for confounding by maternal age, education, ethnicity, income, maternal atopic diseases, parity, smoking status, essential hypertension, infant sex, and birth weight adjusted for gestation and sex, the point estimates for the associations for early wheeze (OR 1.31 [1.10–1.56], *p*=0.002), severe wheeze (OR 1.29 [0.98–1.72], *p*=0.07), and asthma (OR 1.32 [1.09–1.59], *p*=0.004) were slightly attenuated. After adjustment, there was weak evidence for the association with hay fever (OR 1.22 [95% CI 0.97–1.55], *p*=0.09), and eczema (OR 1.12 [95% CI 0.96–1.32], *p*=0.1). Inclusion of birth weight adjusted for gestation and sex did not alter the estimates for gestational hypertension and was not a significant inclusion in any of the models below a *p*-value of 0.15. After additional adjustment for gestational age and caesarean delivery, there remained evidence of an association with early wheeze (OR 1.22 [95% CI 1.02–1.45], *p*=0.03) and asthma (OR 1.20 [95% CI 1.00–1.45], *p*=0.06), although attenuated, but not severe wheeze (OR 1.18 [95% CI 0.89–1.58], *p*=0.3). The full results are shown in Table 2. Population attributable fractions for the total effects of gestational hypertension, calculated from the partially adjusted models, were 2.4% (1.0–3.8%), 0.9% (−0.3% to 2.1%), and 1.8% (0.0 to 3.7%) for asthma, eczema, and hay fever, respectively.

**Table 1:** Baseline characteristics of study participants

Characteristic	Total	PIH/PET	Non-PIH/PET
	(*n* = 12,450)	(*n* = 950)	(*n* = 11,500)
Maternal characteristics			
Age, n. (%)			
<25 years	2926 (22)	219 (21)	2707 (23)
25–29 years	3486 (29)	303 (34)	3183 (28)
30–34 years	3905 (32)	271 (29)	3634 (32)
≥ 35 years	2133 (18)	157 (17)	1976 (18)
Ethnicity, n. (%)			
White	10,836 (90)	854 (92)	9982 (90)
South Asian	1089 (6.3)	55 (4.0)	1034 (6.5)
Black	339 (2.4)	28 (2.8)	311 (2.4)
Other	186 (1.3)	13 (1.3)	173 (1.3)
Household income quintile, n. (%)			
1	2556 (18)	170 (15)	2386 (19)
2	2646 (19)	175 (16)	2471 (20)
3	2457 (20)	200 (22)	2257 (20)
4	2475 (21)	220 (25)	2255 (21)
5	2316 (21)	185 (22)	2131 (21)
Education by NVQ, n. (%)			
None	1912 (14)	105 (11)	1807 (14)
1	979 (8.0)	56 (5.0)	923 (8.3)
2	3604 (30)	305 (33)	3299 (30)
3	1833 (14)	159(16)	1674 (14)
4	3661 (31)	300 (32)	3361 (30)
5	461 (3.7)	25 (2.1)	436 (3.8)
Maternal asthma, n. (%)	2058 (17)	193 (21)	1865 (17)
Maternal hay fever, n. (%)	3046 (26)	271(28)	2775 (25)
Maternal eczema, n. (%)	2186 (19)	208 (24)	1978 (18)
Multiparity, n. (%)	7142 (57)	419 (43)	6723 (58)
Caesarean, n. (%)	2682 (21)	349 (36)	2333 (20)
Infant characteristics			
Male sex, n. (%)	6343 (51)	488 (53)	5855 (51)
Gestation, median weeks (IQR)	40.0 (2.1)	39.0 (2.0)	40.0 (2.0)
Birth weight z-score, mean (SE)	0.33 (0.01)	0.20 (0.04)	0.34 (0.01)
Eczema, n. (%)	4242 (35)	358 (39)	3884 (35)
Asthma, n. (%)	1857 (15)	169 (18)	1688 (14)
Hay fever, n. (%)	1342 (10)	122 (13)	1220 (10)

*PIH* pregnancy-induced hypertension, *PET* preeclampsia, *NVQ* National Vocational Qualification level, *IQR* interquartile range, *SE* standard error

Maternal and infant characteristics according to exposure. Counts are unweighted; proportions, means, and medians are weighted according to MCS wave 3 survey weights

**Table 2:** Odds ratios for primary and secondary exposures with sequential adjustment for potential confounders and potential mediators

Outcome	N. events	Unadjusted ORs (95% CI)	Partially adjusted ORs (95% CI)^1^	Fully adjusted ORs (95% CI) ^2^
Early wheeze	3805	1.34 (1.14, 1.58)	1.31(1.10, 1.56)	1.22 (1.02, 1.45)
Severe wheeze	924	1.31 (0.99, 1.72)	1.29 (0.98, 1.72)	1.18 (0.89, 1.58)
Asthma	1857	1.35 (1.12, 1.62)	1.32 (1.09, 1.59)	1.20 (1.00, 1.45)
Hay fever	1342	1.36 (1.08, 1.71)	1.22 (0.97, 1.55)	1.17 (0.92, 1.48)
Eczema	4242	1.19 (1.02, 1.40)	1.12 (0.96, 1.32)	1.11 (0.95, 1.31)

*OR* odds ratio, *CI* confidence internal

^1^Adjusted for potential confounding by maternal age, education and ethnicity, household income, maternal atopy, parity, smoking status in pregnancy, essential hypertension, infant sex, and birth adjusted for gestation and sex

^2^Additionally adjusted for potential mediation by caesarean delivery and gestational age

### Causal mediation

There was evidence that the association between gestational hypertensive disease and asthma was mediated by gestational age. The estimated total (potential) effect for asthma was comparable to that obtained from the main model after adjustment for potential confounding: OR 1.32 (1.06–1.63, *p*=0.012). The estimated direct effect was OR 1.21 (0.97–1.50, *p*=0.089), in this case comparable to the main model after adjustment for these potential mediators. The estimated indirect effect of gestational age was OR 1.08 (1.05–1.12), with 28% of the association mediated by gestational age. There was no evidence of mediation by gestational age for hay fever or eczema, nor by caesarean delivery for asthma, hay fever, or eczema. The estimated combined indirect, individual indirect, and the direct effects are shown in Table 3.

**Table 3:** Adjusted odds ratios for the decomposed effects of gestational hypertension on the primary outcomes

	**Asthma** **aOR (95% CI)**	**Hay fever** **aOR (95% CI)**	**Eczema** **aOR (95% CI)**
**Total effect**^1^	1.32 (1.06, 1.63)	1.22 (0.96, 1.56)	1.12 (0.95, 1.32)
**Direct effect**^1^	1.21 (0.97, 1.50)	1.17 (0.91, 1.49)	1.11 (0.94, 1.31)
**Indirect effect**^1^	1.09 (1.05, 1.13)	1.05 (1.01, 1.09)	1.01 (0.98, 1.03)
Caesarean^2^	1.01 (0.99, 1.03)	1.02 (0.99, 1.05)	1.01 (0.99, 1.02)
Indirect effect, %	4.7	12	7.1
Gestational age^3^	1.08 (1.05, 1.12)	1.02 (0.99, 1.04)	1.00 (0.98, 1.02)
Indirect effect, %	28	12	1.0

*aOR* adjusted odds ratio, *CI* confidence interval

^1^Total, direct, and indirect effects for both caesarean and gestational age combined, adjusted for maternal age, education and ethnicity, household income, maternal atopic diseases, parity, essential hypertension, smoking status in pregnancy, infant sex, weight by gestational age

^2^Indirect effect of caesarean

^3^Indirect effect of lower gestational age

## Discussion

After adjustment for confounding, there was strong evidence that gestational hypertension was a risk factor for early wheeze and asthma, and weak evidence that it was a risk factor for severe wheeze and hay fever. The effect sizes were weak, with gestational hypertension increasing the odds of early wheeze, severe wheeze, and asthma by approximately a third, and hay fever by a fifth. These partially adjusted findings are relevant because they encompass the potential distal effects of gestational hypertension, including the effects of the timing and mode of delivery. The fraction of cases attributable to this total potential effect of gestational hypertension was minimal. The observed associations were attenuated after further adjustment for potential mediation by caesarean and gestational age. In the formal mediation analysis, 28% of the effect of gestational hypertension was mediated by gestational age, with earlier delivery conferring risk of asthma. The estimated total effect of gestational hypertension in the mediation analysis remained consistent and was weak, and the strength of the evidence for a direct effect of gestational hypertension on asthma was weaker.

The main strengths of this analysis were its sample size, consideration of a comprehensive range of confounders including socioeconomic, maternal health, and pregnancy related sources of confounding, the inclusion of formal mediation analysis and data linking perinatal events and health at age 5. Due to the representativity of the MCS population, the findings are generalizable to a UK population. The main limitation of this study was its use of self-reported exposures and outcomes which may introduce recall bias by under or over-estimating the true disease prevalence. One Canadian validation study found parental report of physician-diagnosed asthma to be highly specific but not sensitive [25], and two validation studies in UK-based cohorts found parental report of physician-diagnosed asthma sensitive and specific compared with GP clinical record of asthma [26, 27]. It is conceivable that parent report of ever having had asthma may be more sensitive but less specific compared with parental report of physician-diagnosed asthma; however, parental report of “having ever had” asthma has shown good agreement with clinical assessment (sensitivity 0.96 and specificity 0.87), the gold standard diagnosis in a Norwegian validation study of 801 children [28]. Additionally, the prevalence of ever asthma in the present study is comparable with that in the English arm of the Mechanisms of the Development of Allergy (MeDALL) cohort which used parental report of physician-diagnosed asthma [29]. The ISAAC questionnaires have also been broadly validated with good sensitivity and specificity [30, 31]. To provide further insight, the symptomatic outcomes supplement the main outcomes with information on recent symptomatic status, “severe wheeze”, and to compare with those who had early wheeze, “early wheeze” with which we also observed strong evidence of an association. Nevertheless, the potential for misclassification varies with the different asthma definitions across population studies. There is mixed evidence on the reliability of self-reported data on gestational hypertension. One Norwegian study on historical pregnancies found self-report to be reliable even among elderly women [32]. In another Danish study, the question “did you have preeclampsia in pregnancy?” had sensitivity 73%, specificity 99%, but a positive predictive value of 59%, and “did you have hypertension in pregnancy?” 85%, 92%, and only 39% respectively, when compared with review of the medical record [33] reflecting a tendency to report disease not truly present. Whilst it is not possible to interpret the effect of the specific mode and wording of the MCS interview, here women were only asked about specific medical problems if they independently recognized a medical problem in the first instance [18]. We theorize that this two-step question may reduce the proportion of women who self-reported gestational hypertensive disease who did not have true disease. Nevertheless, there are likely to be some women who perceived themselves to be hypertensive, potentially following investigation for hypertension and who reported hypertensive disease but who did not have true disease. We believe it to be unlikely that a differential misclassification may have occurred according to atopic disease outcome. Nevertheless, our findings are limited by the use of self-reported data and this misclassification introduced the potential for bias towards the null. Similarly, identifying subtypes of hypertensive disease was not possible. A related limitation was the lack of IgE assay to determine atopic sensitization biochemically which would have enabled more precise classification into atopic or non-atopic disease.

We observed a comparable association between gestational hypertension and early wheeze, severe wheeze, and asthma between the models. The consistency of the association across wheeze types and asthma diagnosis reflects a well-described association [34], and whilst the relationship between gestational hypertension and wheeze up to 2 years has been established [35], the majority of these children do not develop asthma. Early wheeze may signify a viral infective trigger for later asthma or else may be a marker of susceptibility to asthma [34] whilst severe wheeze at age 5 reflects recent and uncontrolled symptoms to provide an alternative construction of asthma. The consistency in the attenuation across these overlapping populations is likely reflective of the related underlying airway disease. However, the risk posed by gestational hypertension appears inconsistent across the different atopic diseases; its differing associations with asthma and eczema in the partially adjusted models as well as the different patterns of potential mediation suggest heterogenous atopic phenotypes. The association of gestational hypertension with asthma and hay fever is congruent with previous findings of an association with asthma and hay fever [36], asthma [37], but also asthma, eczema, hay fever, and food allergy [38]. In the latter registry-based cohort, a dose-dependent association was observed for asthma and hay fever, and only an association between preeclampsia greater than 14 days of duration and eczema [38]. One birth cohort observed no association between gestational hypertension and asthma, although did observe an association with early wheeze and reduced lung function at 8 years [39]. The less consistent findings between atopic diseases in the present study may represent our adjustment for a wider range of confounders but potentially also our use of self-reported and combined PIH and PET.

We considered both the independent effects of gestational age and caesarean in the fully adjusted models, and potential mediation in the mediation analysis. After adjustment for both gestational age and caesarean delivery in the fully adjusted model, there remained evidence of an association between gestational hypertensive disease and asthma. There was only weak evidence to support a direct effect of gestational hypertension on asthma after considering possible mediation by gestational age and caesarean delivery. It would appear that a proportion of the potential effect on asthma is mediated by earlier delivery. There remains weak evidence of a weak effect exerted independently; equally, residual confounding may account for this association. One large registry-based cohort that investigated the relationship between preeclampsia and asthma using formal mediation analysis also found evidence of mediation by gestational age [40]. We have not identified any other formal mediation analyses relating to mode of delivery, nor relating to the outcomes of eczema or hay fever. Whilst it is likely that earlier delivery is a marker of severity of hypertensive disease, prematurity is a recognized risk factor for asthma including in contexts of preterm delivery not related to gestational hypertension [41, 42]. We did not find evidence that gestational hypertension was a risk factor for eczema nor that risk of eczema is mediated in either direction by gestational age or caesarean. This is consistent with findings that prematurity confers risk of asthma but not eczema, or atopic dermatitis, in previous studies [43–45]. The risk of asthma may be modified by efforts to manage risk factors for PIH and PET prior to conception, risk factors for preterm birth more broadly, and prophylactic treatment for PET prevention with aspirin.

Potential mechanisms from gestational hypertension and atopic diseases include the immune dysregulation of pre-eclampsia which may lead to upregulation of placental pro-inflammatory mediators and suppression of anti-inflammatory mediators implicated in atopic disease in offspring [8, 46, 47]. T-helper population imbalance has been found to associate with greater eosinophilia and allergen sensitization [48, 49]. However, complications of gestational hypertension also increase the risk of early delivery [40, 42] and delivery by caesarean section [50, 51]. Preterm infants may be more susceptible to asthma due to physical or immunological lung immaturity, and respiratory secretions of preterm infants have been found to contain pro-inflammatory compounds [52]. Caesarean delivery may alter exposure to skin commensals of the delivering infant which may affect gut microbial composition and therefore immunomodulatory effects of the gut microbiome [53], but we did not find evidence to support the relevance of this pathway in the context of gestational hypertension according to these individual clinical diagnoses. It is difficult to determine whether the observed associations may be due to an in utero effect of pre-eclampsia due to an altered immune environment or whether we may be observing the effects of prematurity. Either way, gestational hypertension was not a strong risk factor for any of the atopic diseases studied. Additionally, we cannot exclude the possibility that the weak association between gestational hypertensive disease and asthma after accounting for mediation is due to residual confounding. Gestational hypertension may be a weak risk factor and a determinant of pregnancy complications which are themselves complex and inter-related; however, within the MCS, we did not observe a significant direct potential effect on the independent clinical entities of asthma, hay fever, or eczema, nor for early wheeze or severe wheeze. If a mechanistic pathway did exist between gestational hypertension and these outcomes, then it accounts only for a minority of cases among women with gestational hypertension. From a public health perspective, the moderate strength of what is an uncommon risk factor means that gestational hypertension, including its distal effects on timing and mode of delivery, is not a major determinant of these diseases as a whole and this was reflected in the small population attributable fractions. We studied these outcomes as individual diagnoses, and it remains possible that gestational hypertension may be a risk factor for specific subtypes of atopic disease.

The decision for delivery in pregnancy complicated by hypertensive disease in general involves balance between reducing maternal morbidity by early delivery and leaving the fetus in utero to prevent the sequelae of preterm birth including respiratory distress syndrome [54]. The obstetrician therefore faces a difficult dilemma when deciding when to deliver a woman with PIH. Substantial respiratory morbidity has been reported in offspring and adults born at 32–36 weeks of age [55], a gestational interval in which obstetricians often consider early delivery for pre-eclampsia. Whilst the risk of childhood asthma is not a consideration when managing the immediate risk hypertensive disease poses to fetus and mother, characterization of the relationship between gestational hypertension and atopic disease and the possible mediating role of earlier delivery provides further insight into the longer-term health of this group of infants.

## Conclusion

We observed a weak association between gestational hypertensive disease and offspring asthma, with part of this relationship mediated by earlier delivery but not by mode of delivery. This study indicates the need to characterize the likely distinct aetiologies of atopic phenotypes further to identify any subtypes of disease with risk factors amenable to modification at the very start of the life course.

## Data Availability

The MCS datasets are available from the UK Data Service

## Abbreviations

CI: Confidence intervals
IgE: Immunoglobulin E
ISAAC: International Study of Asthma and Allergies in Childhood
MCS: Millennium Cohort Study
NVQ: National vocational qualification
OR: Odds ratio
PET: Pre-eclampsia
PIH: Pregnancy-induced hypertension
SD: Standard deviation
SE: Standard error
SES: Socioeconomic status
WHO: World Health Organisation

## Code Availability

N/A
